# Tuftelin1 drives experimental pulmonary fibrosis progression by facilitating stress fiber assembly

**DOI:** 10.1186/s12931-023-02633-w

**Published:** 2023-12-17

**Authors:** Caoyuan Niu, Kai Xu, Yanan Hu, Yanling Jia, Yuexia Yang, Xiaoyue Pan, Ruyan Wan, Hui Lian, Qiwen Wang, Juntang Yang, Yajun Li, Ivan Rosas, Lan Wang, Guoying Yu

**Affiliations:** 1 State Key Laboratory Cell Differentiation and Regulation, Henan International Joint Laboratory of Pulmonary Fibrosis, Henan Center for Outstanding Overseas Scientists of Organ Fibrosis, College of Life Science, Henan Normal University, 46 Jianshe Road, Xinxiang, 453007 Henan China; 2 The Third Affiliated Hospital of Xinxiang Medical University, Xinxiang, China; 3 Division of Pulmonary, Critical Care and Sleep Medicine, Baylor College of Medicine, Houston, TX 77030 USA

**Keywords:** IPF, TUFT1, Stress fiber assembly, N-WASP, Cell activation

## Abstract

**Background:**

Idiopathic pulmonary fibrosis (IPF) is a progressive interstitial lung disease (ILD) with unknown etiology, characterized by sustained damage repair of epithelial cells and abnormal activation of fibroblasts, the underlying mechanism of the disease remains elusive.

**Methods:**

To evaluate the role of Tuftelin1 (TUFT1) in IPF and elucidate its molecular mechanism. We investigated the level of TUFT1 in the IPF and bleomycin-induced mouse models and explored the influence of TUFT1 deficiency on pulmonary fibrosis. Additionally, we explored the effect of TUFT1 on the cytoskeleton and illustrated the relationship between stress fiber and pulmonary fibrosis.

**Results:**

Our results demonstrated a significant upregulation of TUFT1 in IPF and the bleomycin (BLM)-induced fibrosis model. Disruption of TUFT1 exerted inhibitory effects on pulmonary fibrosis in both in vivo and in vitro. TUFT1 facilitated the assembly of microfilaments in A549 and MRC-5 cells, with a pronounced association between TUFT1 and Neuronal Wiskott-Aldrich syndrome protein (N-WASP) observed during microfilament formation. TUFT1 can promote the phosphorylation of tyrosine residue 256 (Y256) of the N-WASP (p^Y256^N-WASP). Furthermore, TUFT1 promoted transforming growth factor-β1 (TGF-β1) induced fibroblast activation by increasing nuclear translocation of p^Y256^N-WASP in fibroblasts, while wiskostatin (Wis), an N-WASP inhibitor, suppressed these processes.

**Conclusions:**

Our findings suggested that TUFT1 plays a critical role in pulmonary fibrosis via its influence on stress fiber, and blockade of TUFT1 effectively reduces pro-fibrotic phenotypes. Pharmacological targeting of the TUFT1-N-WASP axis may represent a promising therapeutic approach for pulmonary fibrosis.

**Supplementary Information:**

The online version contains supplementary material available at 10.1186/s12931-023-02633-w.

## Introduction

Idiopathic pulmonary fibrosis (IPF) is a progressive, chronic, and ultimately fatal interstitial lung disease characterized by enhanced extracellular matrix deposition, repetitive alveolar epithelial injury, dysregulated wound repair, and fibroblasts activation [1, 2]. The median survival of IPF patients without transplants is estimated to be only 2–4 years [3, 4]. Currently, therapeutic choices for IPF remain constrained, with only FDA-approved drugs pirfenidone and nintedanib demonstrating the capacity to modestly decelerate disease progression [5, 6]. Hence, the pursuit of novel preventative and therapeutic modalities stands as a pressing priority in addressing this ailment.

IPF was thought to be a result of multiple genetic and environmental risk factors [7–9]. Identifying the molecular mechanisms underlying the disease and potential prognostic biomarkers is critical for developing effective treatments. In a previous study, a hybrid feature selection method was employed to extract informative genes, with Tuftelin1 (TUFT1) identified as a candidate gene for IPF, while its role in IPF was still unclear [10].

TUFT1 is an acidic protein mainly expressed in ameloblasts, and it plays an essential role in developing and mineralizing tooth tissues [11, 12]. However, it is also expressed in non-mineralizing tissues and has been shown to promote cell proliferation and migration in renal cell carcinoma and metastasis in triple-negative breast cancer and pancreatic cancer [13–15]. Moreover, TUFT1 is regulated by hypoxia and the Hedgehog signaling pathway and is involved in multiple diseases [16]. In NCI-H441 lung adenocarcinoma cells, TUFT1 was shown to be a novel target of transforming growth factor-β1 (TGF-β1) and exhibited a pivotal involvement in the orchestration of microfilament formation within A549 cells [16].

IPF is characterized by the remodeling of the actin cytoskeleton of epithelial cells, fibroblasts and endothelial cells [17]. The actin cytoskeleton is a complex, dynamic biopolymer network that performs essential functions in cell migration, cell interaction with the environment, and mechanical properties of the cell surface [18–22]. The Neuronal Wiskott-Aldrich syndrome protein (N-WASP) is an actin nucleation factor that promotes polymerization of branched actin filaments [23]. The activation form of N-WASP is the phosphorylation of tyrosine residue 256 (Y256) of the N-WASP (p^Y256^N-WASP), which is mediated by TGF-β1 [24].

Our preliminary analysis showed TUFT1 is augmented in IPF lung. We hypothesized that the TUFT1 may be crucial in the pathogenesis of the disease. Herein we aimed to investigated the role of TUFT1 in IPF and evaluate the role of TUFT1 in IPF and elucidate its molecular mechanism. We investigated the level of TUFT1 in the IPF and bleomycin-induced mouse models and explored the influence of TUFT1 deficiency on pulmonary fibrosis. Additionally, we explored the effect of TUFT1 on the cytoskeleton and illustrated the relationship between stress fiber and pulmonary fibrosis.

## Materials and methods

### Reagents and antibodies

Recombinant human TGF-β1 was obtained from R&D systems (Minneapolis, MN, USA). Bleomycin sulfate was obtained from Hisun Pharm (Taizhou, China). Sodium orthovanadate (SOV) was obtained from Sigma-Aldrich (Shanghai, China). Wiskostatin (Wis) was obtained from Abcam (Shanghai, China). N-WASP was obtained from Proteintech (Wuhan, China). Goat anti-rabbit IgG of β-actin, Alpha-smooth muscle actin (α-SMA), p^Y256^N-WASP and GAPDH were purchased from Affinity Biosciences (Changzhou, China). Goat anti-mouse IgG of Vimentin and E-cadherin were purchased from Cell Signaling Technology (Shanghai, China), Goat anti-rabbit IgG of N-cadherin, Fibronectin and Collagen I were purchased from Cell Signaling Technology (Shanghai, China), TUFT1 was purchased from Thermo Fisher (Suzhou, China).

### Human Lung tissues

Human lung tissues were obtained by surgical lung biopsy (SLB) from patients in Xinxiang central hospital**,** IPF was diagnosed according to the international guidelines [25]. The human lung tissue study was approved by the Xinxiang central hospital Medical Research Ethics Committee (No.2019-01-12) and was conducted in accordance with the Declaration of Helsinki. All patients who donated tissues have provided informed consent.

### Mice

All institutional and national guidelines for the care and use of laboratory animals were followed. Animal maintenance and handling procedures was approved by Henan Normal University Institutional Animal Care and Use Committee (IACUC, SMKX-2118BS1018). Wild-type male C57BL/6N mice (9–10 weeks, 19–24 g) were obtained from the Charles River (Beijing, China). Mice were raised in a controlled environment with a cycle of 12-h light/dark, auto-regulated temperature and humidity, and access to food and water unrestricted. To knock down the *Tuft1* gene in vivo, The mice were anesthetized by isoflurane and followed by challenge with type2/6 Adeno-associated virus (AAV2/6) adenoviral particles containing plasmid vector (pcDNA3.1-U6-shTuft1-CMV-ZsGreen, sequence: 5′-TGAAGAGATCATTAAGGTTTA-3′) (50 μL, 1.8 × 1012 Vg/mL, i.t.) or equivalent adenoviral particles loaded with none-target control vector (pcDNA3.1-U6-shNC-CMV-ZsGreen, sequence: 5′-TTCTCCGAACGTGTCACGTAA-3′) (purchased form HANBIO, Shanghai, China) 7 days before the Bleomycin (BLM) challenge. For fibrosis inducing, the mice were anesthetized by isoflurane and bleomycin (50 µL, 1.5 U/kg.bw, i.t.) or equivalent 0.9% saline. Finally, the mice were euthanized with 20% Ethyl Carbamate (800 mg/kg, i.p) (Sigma-Aldrich, Missouri, USA) and lung samples were collected on day 14 after BLM challenge.

### Cell culture

The human lung adenocarcinoma cell line A549 (ATCC^®^ CCL-185™, ATCC, Manassas, VA), negatively tested for mycoplasma, was cultured in DMEM/F12 medium supplied with 10% fetal bovine serum and antibiotics (100 units/mL penicillin and 100 μg/mL streptomycin). The human embryonic lung fibroblast cell line (MRC-5) was purchased from Procell Life Science &Technology Co., Ltd. (Wuhan, China) and cultured in MEM medium (containing NEAA) (Shanghai Zhong Qiao Xin Zhou Biotechnology Co., Ltd., ZQ-300) supplied with 10% fetal bovine serum and antibiotics (100 units/ml penicillin and 100 μg/ml streptomycin) in an atmosphere of 5% CO_2_ and humidified at 37 °C. For TGF-β1 stimulation, cells were serum-starved overnight before 5 ng/mL TGF-β1 was added for 24 h.

### Small interfering RNA (siRNA) transfection

TUFT1/siRNA-1(5′-GGAGTCCCATGATGGACAT-3′), TUFT1/siRNA-2(5′-GCAGAGGCUGUGUGACAAATT-3′), TUFT1/siRNA-3(5′-GAGGAACTTCGGAGCAACA-3′) were purchased from RIBOBIO (Guangzhou, China). To transfect siRNA into A549 and MRC-5 cells, the cells were cultured on 6-well plates at 50–70% coverage before transfection. Lipofectamine RNAi MAX (RIBOBIO, Guangzhou, China), Opti-MEM (Thermo Fisher) and Individual siRNA (50 nM) were mixed completely and incubated for 10 min at room temperature. The efficiency of transfection was testified by western blot in 48 h after transfection.

### Micro-CT scanning

Mice from each group were anesthetized by isoflurane and fixed in the supine position. The pulmonary CT images of each mouse were photographed by a CT imaging system (SkyScan 1276, Bruker, Kontich, Belgium) at the 14th day. And the parameters were set as follows: 65 kV, 200μA, over a total angle of 360◦ for a total scan time of approximately 15 min, and the micro-CT image acquired with a respiratory-gated technique. Micro-CT images of pulmonary were reconstructed by using insta-Recon software (Bruker, Kontich, Belgium).

### Hydroxyproline assay

A hydroxyproline assay kit (MAK008, Sigma, St. Louis, MO, USA) was used to detect the content of hydroxyproline in mouse right lung [26]. Briefly, the fresh lung tissue was homogenized in water (100 µL water of every 10 mg lung tissue), followed by hydrolyzing the samples after adding equivalent 12 N HCl for 3 h at 120 °C. Next, the samples were centrifuged (12,000 rpm, 4 °C, 3 min; Beckman Microfuge 20R) and extracted 10 µL supernatant of every sample, the samples were transferred to a 96-well plate and evaporated at 56 ℃. The samples in the plate were incubated for 5 min at 20 ℃ after added 100 µL chloramine T reagent into each well. Afterward, the samples were incubated for 90 min at 56 °C after mixed with 100 µL p-dimethylaminobenzaldehyde reagent. Absorbance of samples was measured by BioTek ELx800 plate reader at λ = 560 nm (Winooski, VT, USA). The hydroxyproline content of samples was calculated by a standard sample and presented as µg hydroxyproline per right lung.

### Histological and morphometric analysis

The lungs of mice were collected and fixed with 4% paraformaldehyde. The lung tissues were embedded in paraffin and sliced into 3 μm sections. The sections were stained with Masson’s trichrome (Beyotime, Guangzhou, China) and H&E (Beyotime, Guangzhou, China) to assess the collagen content and evaluate the morphometric changes. The acquisition of panoramic images was performed using a BioTek Cytation C10 Confocal Imaging Reader. Images were analyzed by Image Pro Plus software (version 6.0, Media Cybernetics). In order to devise an adapted Ashcroft score, five fields from each lung section were randomly chosen under 100× magnification. The mean score for each field was determined independently by two pathologists in a blinded fashion [27].

### Immunohistochemical (IHC) staining

The lung slides were prepared following previously described protocols [28]. The lung sections were incubated with the primary antibodies overnight at 4 °C, then the secondary antibody biotinylated anti-rabbit IgG (1:100, Beyotime, Guangzhou, China) was incubated for 60 min at 37 °C. Followed by incubating with SABC (1:100, Beyotime, Guangzhou, China), and then visualized by DAB stain (Beyotime, Guangzhou, China). Cell nuclei were stained with hematoxylin.

### Wound-healing assay

SiRNA control or TUFT1 siRNA was transfected into A549 and MRC-5 cells. After 24 h, the cells were placed in a 6-well plate using a culture medium containing 1% FBS. Upon reaching confluence, a pipette tip was utilized to create a direct scratch across the cell monolayer. Pictures were taken at the predetermined time intervals to document the progression of wound healing. Wound healing was calculated as follows: [1−(Width of the wound at a given time/width of the wound at t = 0)] × 100%.

### Transwell assay

Cell migration assay was performed using a transwell chamber (Corning Incorporated, Corning, NY, USA, cat:3422). A549 cells transfected with SiRNA control or TUFT1 siRNA were seeded into transwell plates with serum-free medium, then medium containing 10% FBS was added to the bottom of the chamber. After 24 h, Migration cells were fixed and stained with 1% crystal violet and counted in three random fields under an optical microscope.

### Collagen gel contraction assay

Collagen gel contraction assay was performed using a cell contraction assay kit (CBA-201, Cell Biolabs, Inc., San Diego, CA, USA). MRC-5 cells were suspended in bovine type 1 collagen solution (chilled on ice) at the concentration of 1.85 mg/mL and seeded at a density of 1 × 10^6^ cells/well in a Costar low attachment surface polystyrene 24-well plates (Corning Incorporated, Corning, NY, USA). The plates were then placed in a 37 °C incubator for 1 h to allow for collagen gel polymerization, and 1 mL culture medium was added on top of the gel lattice, and cells were incubated overnight. Then cells were pre-incubated with sodium orthovanadate (20 µM) for 24 h or Wiskostatin (10 µM) for 1 h. Once the contraction agonists were added, the gels were meticulously detached from the sidewalls of each well by a sterile scalpel. Photographs were captured after 24 h, and the analysis of gel disc area was conducted using Image J software for quantification.

### Immunofluorescence staining

The 4% paraformaldehyde was used to fix the lung sections and cell slides and 0.3% Triton X-100 was used to elevate the permeable ability of cells. Then, the sections were block by 10% goat serum for 0.5 h at 37 °C and incubated with primary antibodies overnight at 4 °C. Corresponding secondary antibodies were incubated for 1 h at 37 °C. F-actin was stained with Phalloidin-iFluor 594 Reagent (ab176757, Abcam, USA) and nuclei were stained with DAPI. The images were captured by a fluorescence microscope (Zeiss LSM780, Carl Zeiss).

### Western blotting

The steps of Western blot analyses were described as previously [29]. Initially, lung homogenates or cell lysates were prepared by RIPA lysis buffer (AR0102, Boster, Wuhan, China). The protein concentrations were detected by BCA kit (Solarbio, Beijing, China). The protein was loaded onto SDS-PAGE followed by electrotransferred onto PVDF membranes completely. The membranes were incubated with primary antibodies overnight at 4 °C and incubated with corresponding secondary antibodies for 1 h at room temperature. The images were captured by Odyssey Imaging System (LI-COR Biosciences, Lincoln, NE, USA). The densitometry band quantification was measured by the Image Studio software (LI-COR Biosciences, NE, USA).

### Co-immunoprecipitation (co-IP)

HEK293T cells were lysed in co-immunoprecipitation buffer (AR0107, Boster, Wuhan, China) and incubated for 30 min on ice. The lysate was then centrifuged at 10,000 ×*g* for 10 min and the supernatant was collected. The supernatant was incubated with anti-HA beads (Bimake, B26202, Shanghai, China) or anti-Flag beads (B26102, Bimake, Shanghai, China) at 4 °C overnight. The beads were washed three times with immunoprecipitation wash buffer (50 mM Tris, 150 mM NaCl, 0.5% Tween 20, pH 7.5) and subjected to further analysis.

### Statistical analysis

The statistical analysis involved using a two-tailed Student’s t-test for comparing two experimental groups. One-way or two-way analysis of variance was used for multi-group comparisons, followed by Tukey’s multiple comparison test. Differences were considered statistically significant in all cases when *P* < 0.05.

## Results

### TUFT1 was increased in lungs of IPF patients and bleomycin induced mouse lung fibrosis

**Fig. 1:**
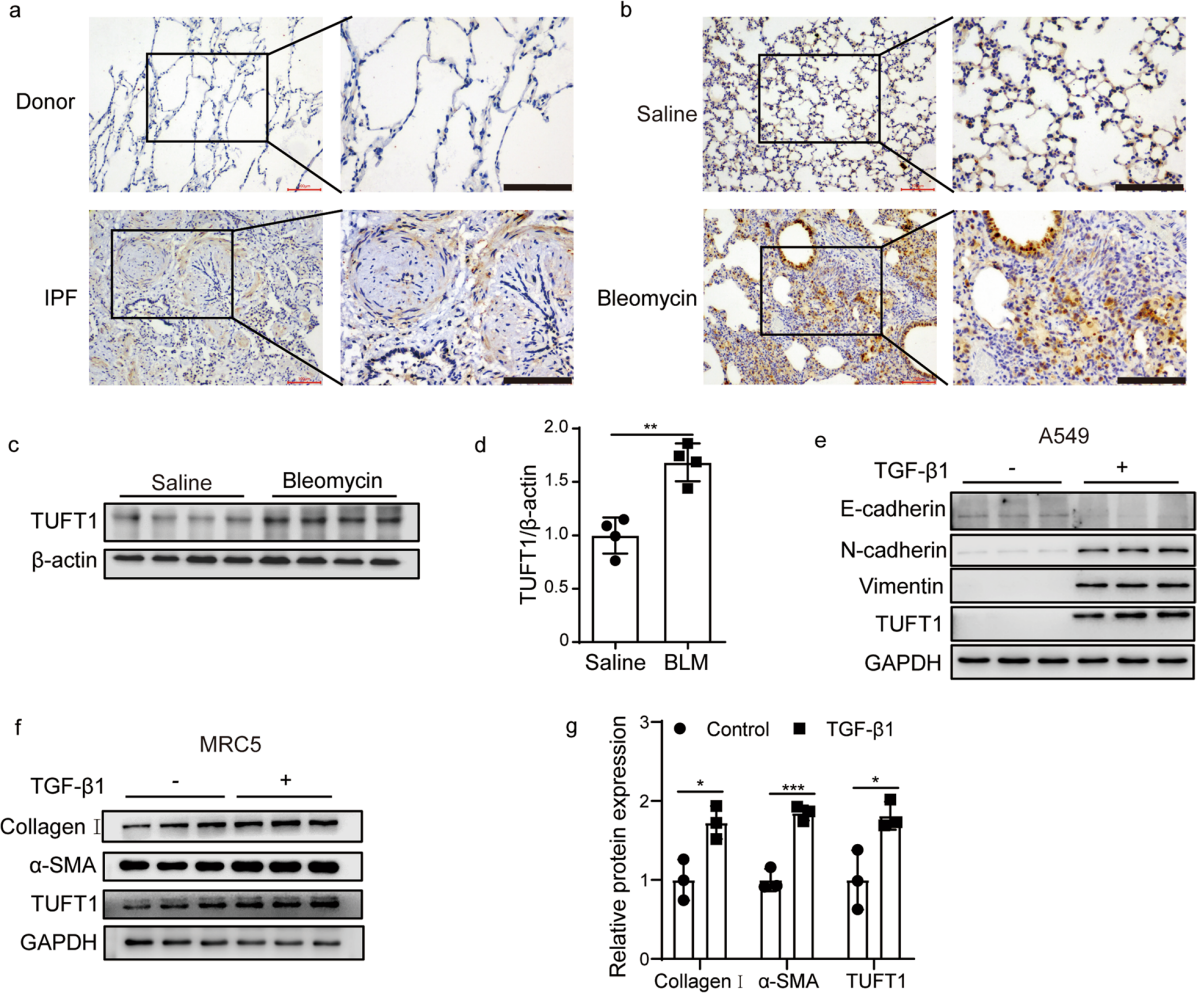
TUFT1 was upregulated in lungs of IPF and BLM‑induced fibrotic mouse lungs. **a** Representative image of immunohistochemistry staining of TUFT1 in the lung sections from the Donor and IPF patient, n = 3 patients or donors, scale bars:100 µm. **b** Representative image of immunohistochemistry staining of Tuft1 in bleomycin induced fibrotic lung, n = 3 mouse per group, scale bars:100 µm. **c** The Tuft1 protein level in lung homogenates from bleomycin-induced fibrotic models was determined using Western blot analysis, n = 4 mouse per group. **d** Densitometry analysis of fig c (mean ± SD). **e** E-cadherin, N-cadherin, Vimentin and TUFT1 were determined by Western blot in A549 cells induced by TGF-β1, GAPDH used as loading control. **f** TUFT1, collagen I and α-SMA protein expression determined by Western blot in TGF-β1-induced MRC-5 cells, GAPDH used as loading control. **g** Densitometry analysis of fig f (mean ± SD). *P < 0.05, **P < 0.01, ***P < 0.001, and ****P < 0.0001

IHC showed that TUFT1 was increased significantly in IPF lung tissues, and TUFT1 was mainly located in myofibroblasts in the fibroblast foci of the IPF lung (Fig. 1a). Meanwhile, we detected the expression of Tuft1 in bleomycin induced fibrotic mouse lung tissue. Consistent with IPF lung, Tuft1 was increased significantly in BLM-induced fibrotic mouse lungs (Fig. 1b–d). To further establish the association between TUFT1 and pulmonary fibrosis in an in vitro context, A549 and MRC-5 cells were exposed to TGF-β1 (5 ng/mL) for a duration of 24 h. This treatment resulted in a marked reduction in the epithelial marker E-cadherin, coupled with a pronounced elevation in the mesenchymal markers N-cadherin and Vimentin. Furthermore, the protein levels of TUFT1 were notably upregulated in TGF-β1-induced A549 cells (Fig. 1e). Similarly, the ECM protein collagen I and myofibroblasts marker α-SMA increased in TGF-β1-treated human lung fibroblasts (MRC-5 cells), and the protein levels of TUFT1 were increased in TGF-β1-induced MRC-5 cells (Fig. 1f, g). These results sustain the idea that augmented TUFT1 may have clinically relevant implications in pulmonary fibrosis.

### Loss of TUFT1 alleviated bleomycin-induced mouse lung fibrosis

**Fig. 2:**
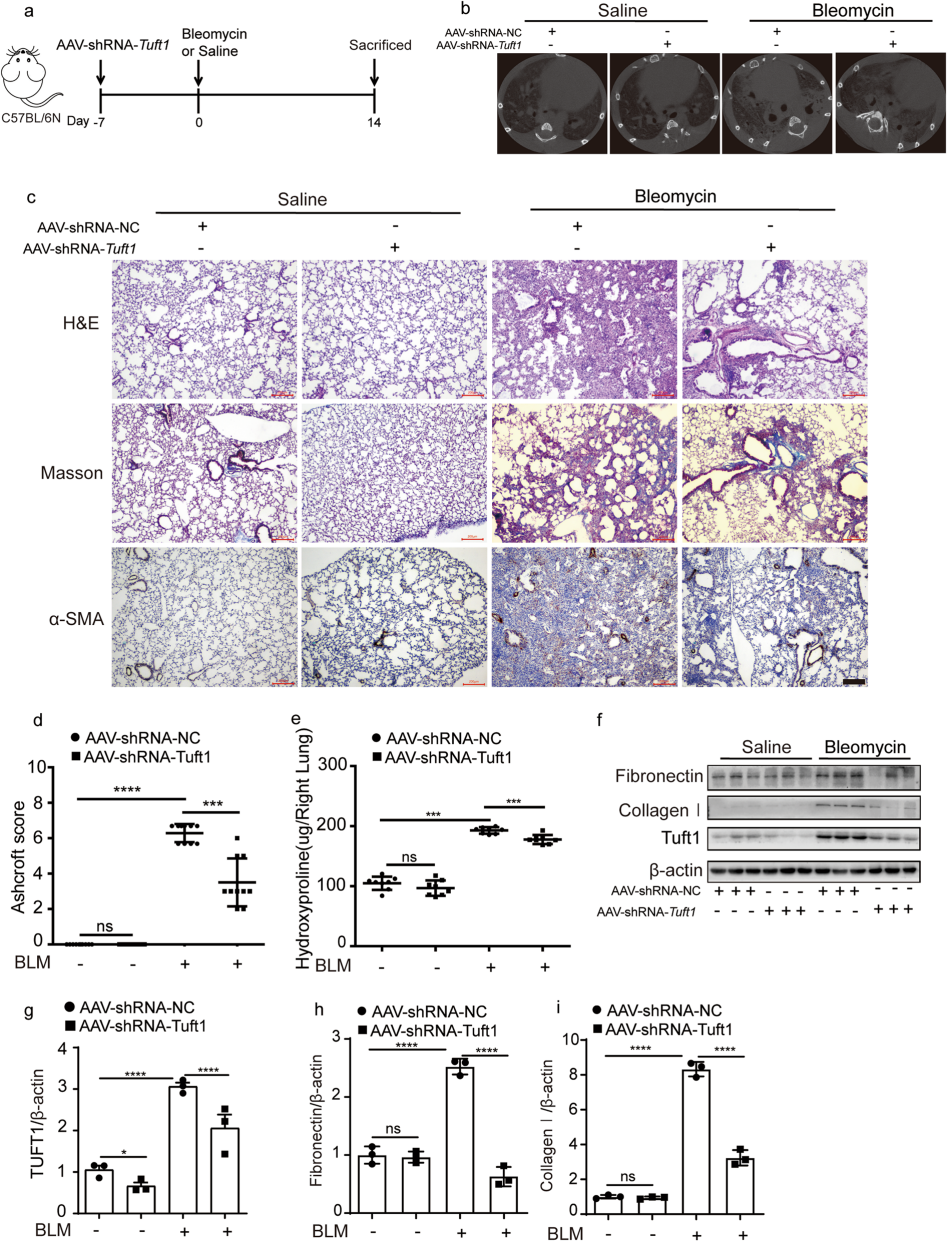
TUFT1 disruption inhibited bleomycin-induced mouse pulmonary fibrosis in vivo. **a** A schematic diagram illustrated the time and course of the experiments. **b** The pulmonary images of mice were detected by Micro-CT scan on the 14th day. **c** Representative images of H&E staining, Masson’s trichrome staining of the lung section, and Immunohistochemical staining images of α–SMA, scale bars: 200 µm. **d** Ashcroft score (mean ± SD). **e** Quantitative hydroxyproline assay of the right lung (mean ± SD, n = 8 mice per group). **f** Western blotting assay of FN, collagen I, and Tuft1 in the lung homogenate; β-actin used as the loading control. **g**-**i** Corresponding optical densitometry analysis of (**f**) (mean ± SD). *P < 0.05, **P < 0.01, ***P < 0.001, and ****P < 0.0001

To investigate the role of TUFT1 in pulmonary fibrosis, we examined fibrotic responses after the silenced TUFT1 expression by using Adeno-associated virus knockdown of TUFT1 (*Tuft1*/short hairpin RNA (shRNA)) in bleomycin-induced mouse pulmonary fibrosis model. The schematic diagram illustrated the time and course of the experiments in Fig. 2a. Micro-CT images taken on day 14 showed the bleomycin treatment caused an obvious density increase in mouse lungs, and the *Tuft1*/shRNA groups showed a marked reduction in the lung density indicated by increased parenchymal opacity (Fig. 2b). Moreover, mice treated with *Tuft1*/shRNA adenovirus showed normal lung tissues structure compared with the bleomycin group as detected by H&E staining (Fig. 2c). Meanwhile, the Masson’s trichrome staining showed that the collagen deposition increased obviously in bleomycin groups, whereas this phenomenon could be rectified by disruption of TUFT1 (Fig. 2c), furthermore, silencing the Tuft1 reduced the expression of α-SMA as indicated by immunohistochemistry staining (Fig. 2c). Ashcroft histopathological grading score was calculated according to hologram of the Masson’s trichrome staining (Additional file 1: Fig. S1), bleomycin treated mice showed increased Ashcroft histopathological grading score compared to saline group, while silencing the Tuft1 decreased the Ashcroft score significantly (Fig. 2d). As expected, the bleomycin treatment enhanced the level of Hydroxyproline sharply, and the *Tuft1*/shRNA alleviated this phenomenon (Fig. 2e). Meanwhile, the levels of Fibronectin and collagen I was decreased by *Tuft1*/shRNA in pulmonary fibrosis process (Fig. 2f–i). Interestingly, the Phalloidin staining revealed a marked abundance of F-actin within the fibrotic tissues of the bleomycin-induced lung fibrosis model, whereas the *Tuft1*/shRNA led to a reduction in F-actin formation in bleomycin-challenge lung tissues (Additional file 1: Fig. S2). Altogether, these data indicate that inhibition of TUFT1 alleviated bleomycin-induced experimental models of lung fibrosis.

### Blockade of TUFT1 inhibited fibrotic phenotype of epithelial cells and fibroblasts in vitro

**Fig. 3:**
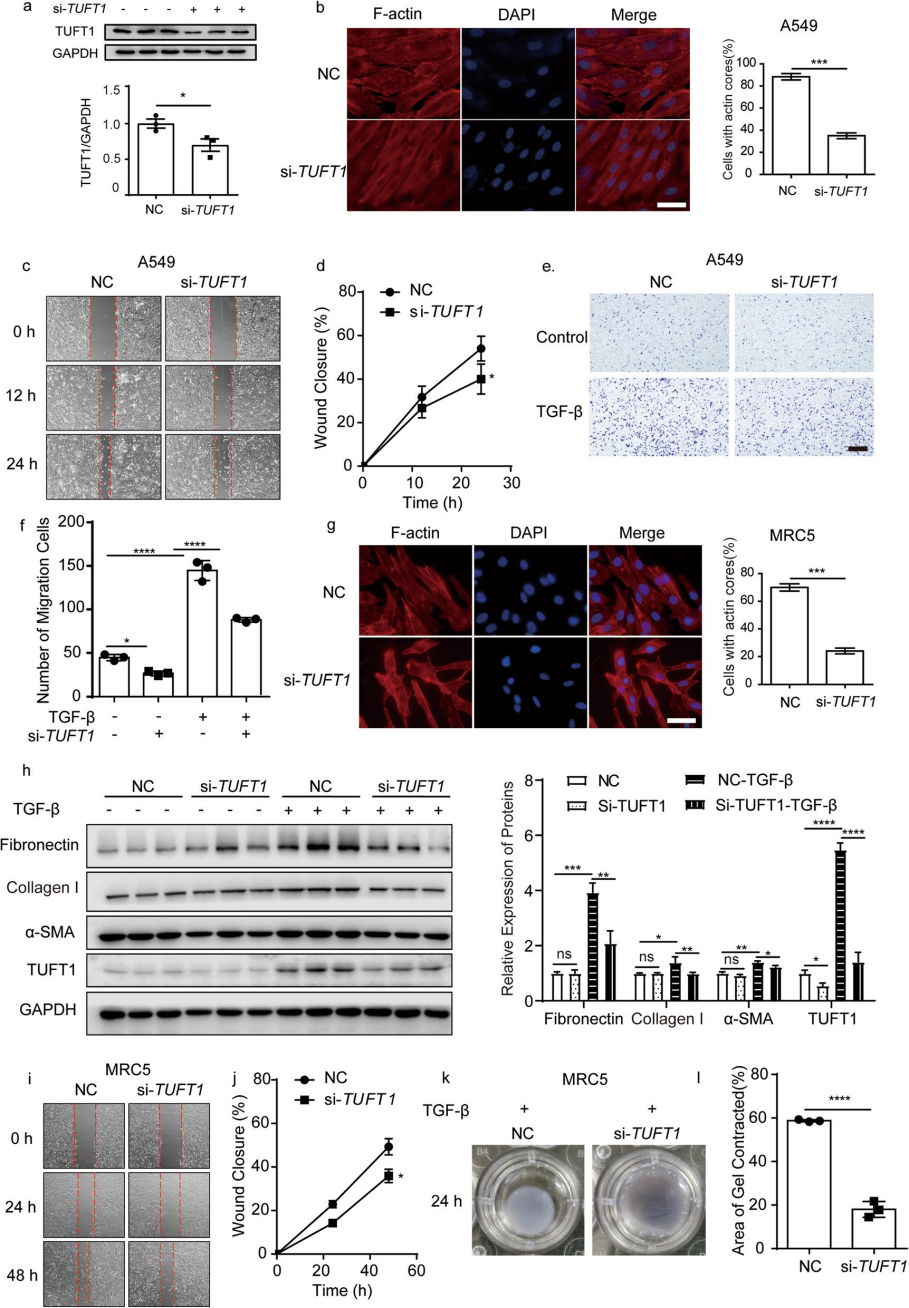
Blockade of TUFT1 inhibited TGF-β1-induced fibrosis in vitro. **a** Interference efficiency of *TUFT1* specified siRNA in the A549 cells detected by Western blotting. **b** The microfilaments morphology of the A549 cells was revealed by the phalloidin staining in si-TUFT1 groups and control, scale bars: 10 µm. F-actin was quantified by the percentage of the cells with the actin cores. **c**, **d** The wound healing progression of A549 cells in si-TUFT1 groups and control. **e**, **f** The migration ability of A549 cells in si-TUFT1 groups and control, scale bars: 1 mm. **g** The microfilaments morphology of the MRC-5 cells was revealed by the phalloidin staining in si-TUFT1 groups and control, scale bars: 100 µm. F-actin was quantified by the percentage of the cells with the actin cores. **h** The expression of FN, collagen I, and α-SMA proteins were determined by Western blot after the silence of TUFT1 in TGF-β1-induced MRC-5 cells. **i**, **j** The wound healing progression of MRC-5 cells in si-TUFT1 groups and control. **k**, **l** The activation of fibroblasts was examined based on fibroblast contraction in 3D collagen matrices (n = 3 independent experiments). ImageJ software was used to measure the area of gels. *P < 0.05, **P < 0.01, ***P < 0.001, and ****P < 0.0001

Next, we intend to explore the influence of TUFT1 on the fibrotic phenotype of epithelial cells and fibroblasts in vitro. Knockdown efficiency of silencing TUFT1 in A549 cells was evaluated by Western Bolt (Fig. 3a). Phalloidin staining revealed that the silencing of TUFT1 resulted in distinct cell morphology changes in TGF-β1 treated A549 cells (Fig. 3b). Moreover, silencing TUFT1 impaired the migration ability of TGF-β1 treated A549 cells (Fig. 3c, d). Meanwhile, the knockdown of TUFT1 also reduced the invasion of TGF-β1 treated A549 cells detected by transwell (Fig. 3e, f). In parallel, we also found that the silencing of TUFT1 influences the microfilaments morphology in TGF-β1 treated MRC-5 cells detected by phalloidin staining (Fig. 3g). Moreover, the knockdown of TUFT1 could decrease the expression of Fibronectin, collagen I, and α-SMA in TGF-β1 treated MRC-5 cells (Fig. 3h). Also, silencing TUFT1 impaired the cell migration of the MRC-5 cells post the induction of TGF-β1 (Fig. 3i, j). Finally, we found that knocking down TUFT1 decreased the contraction ability of TGF-β1 treated MRC-5 cells (Fig. 3k, l). These data implicated that disruption of TUFT1 suppresses the activation of both epithelial cells and fibroblasts in vitro.

### N-WASP is indispensable in TUFT1-mediated pro-fibrotic phenotype

**Fig. 4:**
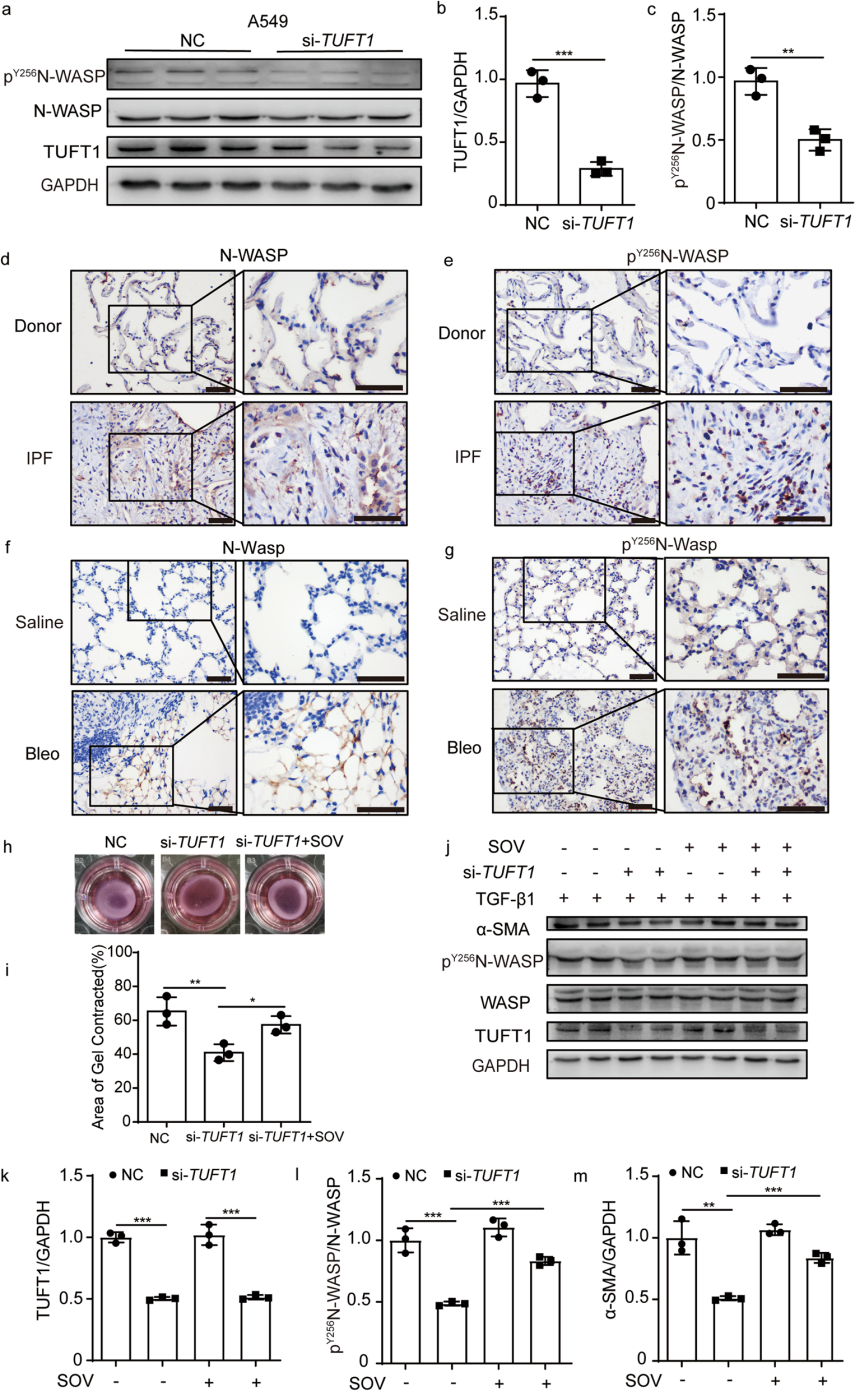
N-WASP played an important role in the progress of TUFT1 interfering with fibrosis. **a**–**c** Knockdown of TUFT1 can inhibited the phosphorylation level of N-WASP in A549 cells. **d** Immunohistochemical staining of N-WASP in IPF lung tissue compare to the donor. **e** Immunohistochemical staining of p^Y256^N-WASP in IPF lung tissue compare to the donor. **f** Immunohistochemical stain images of N-Wasp in bleomycin induced fibrosis lung tissue compare to the control. **g** Immunohistochemical stain images of p^Y256^N-Wasp in bleomycin induced fibrosis lung tissue compare to the control, scale bars: 50 µm. **h**, **i** Silence the TUFT1 in MRC-5 cells could slow down the matrigel contraction compare to the control, and the sodium orthovanadate could reverse this phenomenon. **j** Silence the TUFT1 could decrease the expression of p^Y256^N-WASP and α-SMA, the sodium orthovanadate could relieve the decrease level of p^Y256^N-WASP and α-SMA in TGF-β1 treated MRC-5 cells. **k**–**m** Corresponding optical densitometry analysis of J (mean ± SD). *P < 0.05, **P < 0.01, and ***P < 0.001

As silencing of the TUFT1 distorted the shape of microfilaments, in order to illustrate the mechanism of TUFT1 influence the microfilaments, we examined the expression level of N-WASP and p^Y256^N-WASP, which influenced the assembly of cell microfilaments. The p^Y256^N-WASP was decreased significantly by silencing TUFT1 in TGF-β1 treated A549 cells (Fig. 4a–c). Interestingly, N-WASP and p^Y256^N-WASP were augmented in IPF lungs compared with the non-IPF donor as indicated by immunohistochemistry staining (Fig. 4d, e). Consistently, N-WASP and p^Y256^N-WASP were increased significantly in the bleomycin-induced fibrosis mice model compared to the control (Fig. 4f, g).

Next, we aimed to ascertain whether the p^Y256^N-WASP was involved in the TUFT1-mediated pro-fibrotic phenotype. Sodium orthovanadate (20 µM), a phosphorylase inhibitor of Tyrosine, was used to interfere with the dephosphorylation of p^Y256^N-WASP after the silenced of TUFT1 for 48 h. The result indicated that sodium orthovanadate reversed the impaired contract ability of TGF-β1 treated MRC-5 cells transfected with si-TUFT1 (Fig. 4h, i). Moreover, silencing TUFT1 decreased the level of p^Y256^N-WASP in TGF-β1 treated MRC-5 cells, as well as the myofibroblast marker α-SMA, and the sodium orthovanadate reversed the decrease of both p^Y256^N-WASP and α-SMA (Fig. 4j–m).

### TUFT1 interacted with N-WASP and mediated the phosphorylation of N-WASP

**Fig. 5:**
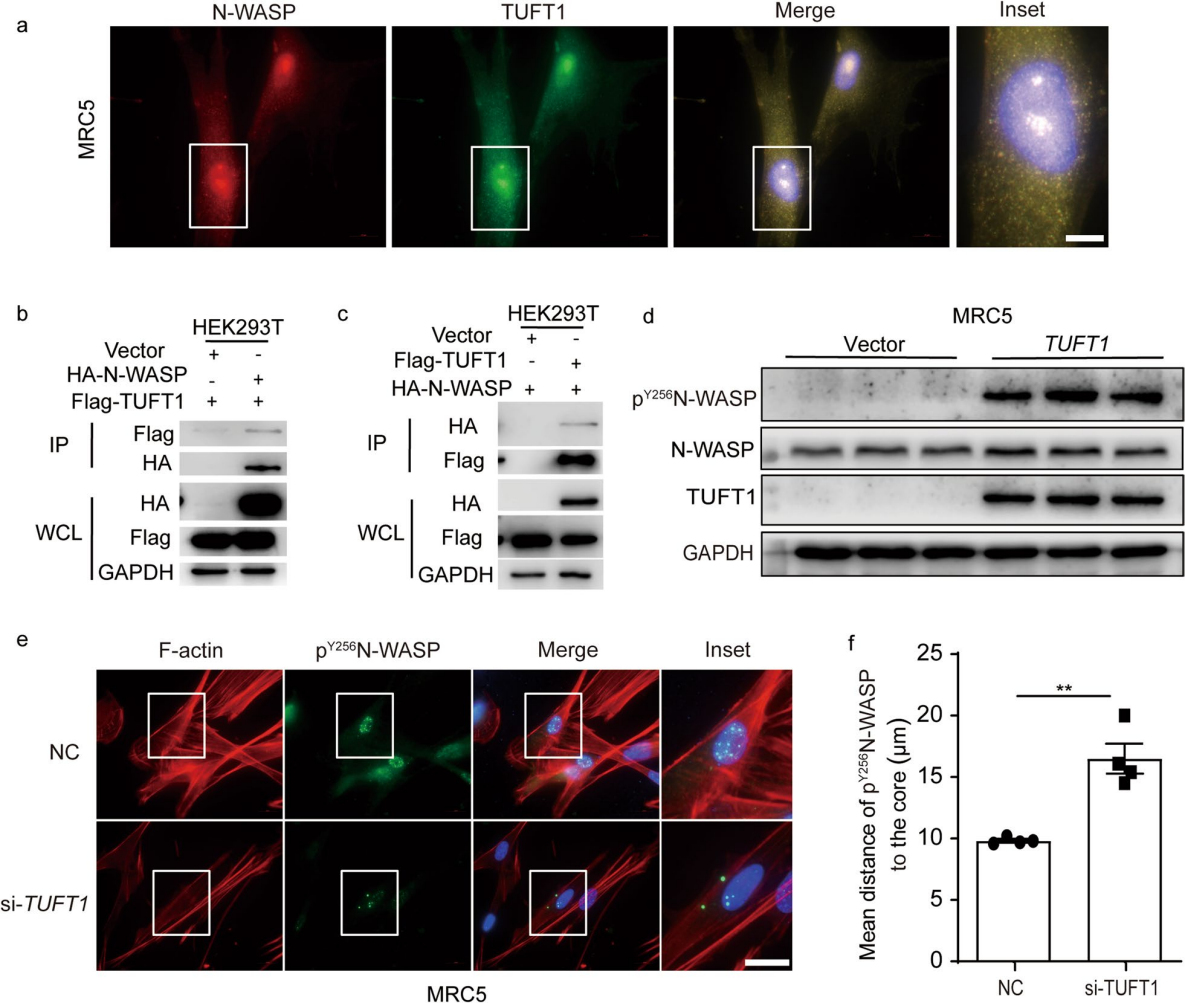
TUFT1 interacted with N-WASP and affected the expression of p^Y256^N-WASP. **a** N-WASP and TUFT1 were co-localized in MRC5 cells detected by immunofluorescent staining, scale bars: 10 µm. **b** Exogenously expressed N-WASP and TUFT1 co-immunoprecipitated in HEK293T cells. **c** Exogenously TUFT1 and N-WASP have fine co-immunoprecipitated in HEK293T cells. **d** TUFT1 can induce a high expression of p^Y256^N-WASP in MRC5 cells. **e** Silencing the TUFT1 could make the p^Y256^N-WASP keep away from the nucleus in MRC5 cells compared to the control detected by immunofluorescent staining, scale bars: 20 µm. **f** The distance of p^Y256^N-WASP to the core of the nucleus was quantified

To explore the relationship between TUFT1 and N-WASP in epithelial cells, we constructed a vector transiently expressing N-WASP. Over-expressed N-WASP led to a substantial increase in TUFT1 fluorescence intensity in MRC5 cells, N-WASP and TUFT1 were co-localized in the cytoplasm (Fig. 5a). Furthermore, exogenously expressed N-WASP and TUFT1 were co-immunoprecipitated suggesting a physical interaction between them (Fig. 5b, c). Likewise, over-expressed TUFT1 also induced the accumulation of phosphorylated N-WASP in MRC5 cells (Fig. 5d). Interestingly, while TGF-β1-induced p^Y256^N-WASP accumulated in the cell nucleus, silencing TUFT1 led to a dispersed distribution of p^Y256^N-WASP in the cytoplasm, and this phenomenon coincidence with the microfilament disruption induced by silencing TUFT1 in MRC-5 cells (Fig. 5e, f). A similar scenario was observed in A549 cells (Additional file 1: Fig. S3). Collectively, these above results showed that TUFT1 interacts with N-WASP and influenced the level and distribution of p^Y256^N-WASP.

### TUFT1 mediates stress fiber formation in a p^Y256^N-WASP dependent manner

**Fig. 6:**
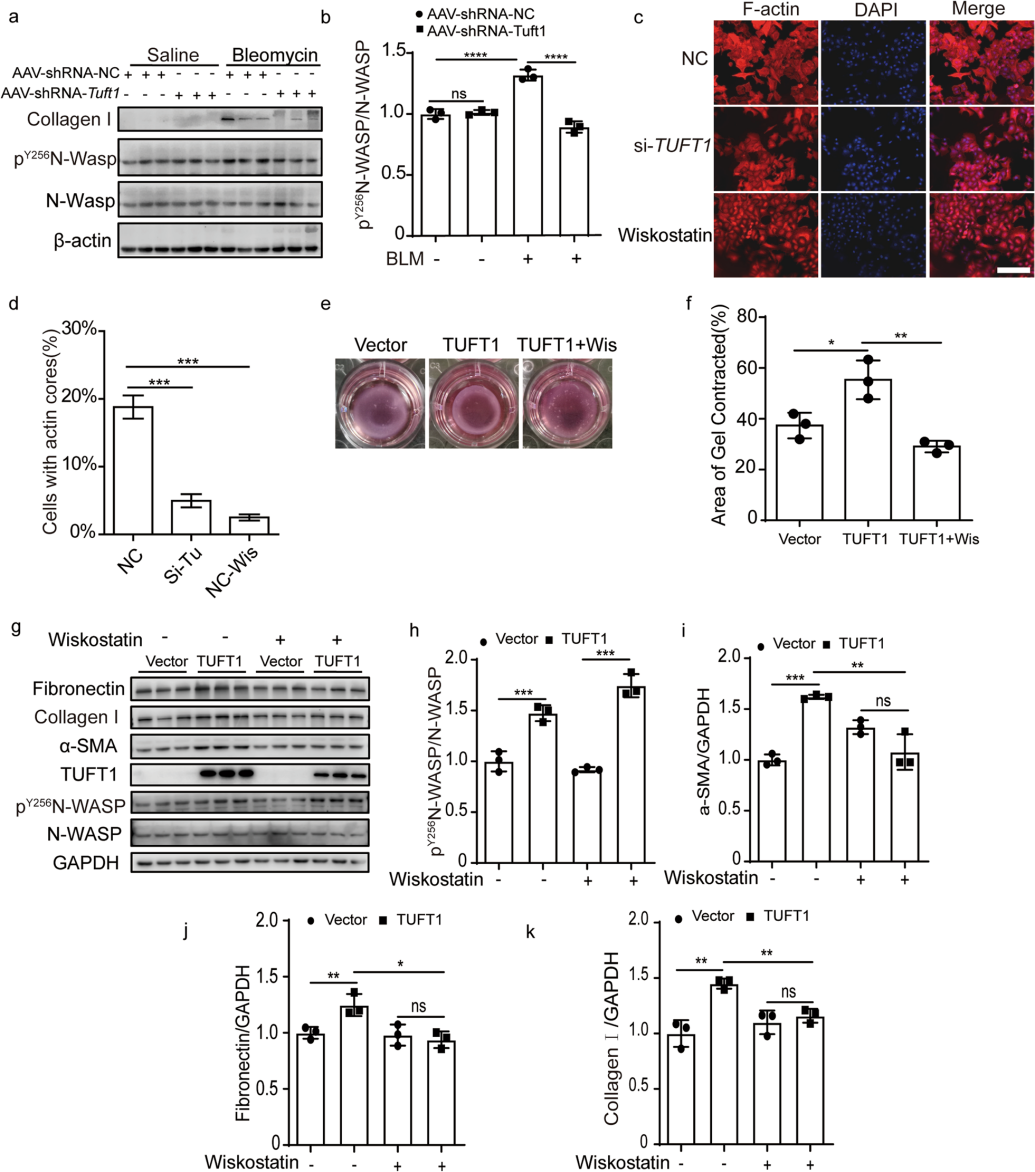
TUFT1 mediated lung fibrosis in a p^Y256^N-WASP-dependent manner. **a** Western blotting assay of p^Y256^N-WASP, N-WASP, and collagen I in the lung homogenate; β-actin used as the loading control. **b** The relative expression of p^Y256^N-WASP/N-WASP. **c** The expression of F-actin in the si-TUFT1 group, wiskostatin group, and control detected by Phalloidin staining in A549 cells, scale bars: 200 µm. **d** The percentage of cells with actin cores in A549 according to (**c**). **e**, **f** Over-expressed TUFT1 in MRC-5 cells could accelerate the matrigel contraction, and Wiskostatin could delay this phenomenon. **g** Over-expressed TUFT1 induced expression of p^Y256^N-WASP and fibrosis marker FN, collagen I, and α-SMA, while wiskostatin could reverse this phenomenon detected by western-blot. **h**–**k** Corresponding optical densitometry analysis of (**g**) (mean ± SD). *P < 0.05, **P < 0.01, ***P < 0.001, and ****P < 0.0001

N-WASP was known to play a critical role in regulating the formation of α-SMA-containing cytoplasmic filaments during myofibroblast differentiation and contractility through active the ARP2/3 complex [24]. We found phosphorylation of N-Wasp at Y256 was induced in BLM-challenged fibrotic lung, whereas silencing the Tuft1 could decrease the level of phosphorylated N-Wasp and collagen I in BLM-challenged fibrotic lung (Fig. 6a, b). Meanwhile, we found silencing the Tuft1 could disperse the p^Y256^N-Wasp in the fibrotic areas (Additional file 1: Fig. S4).

TUFT1 was engaged in the formation of microfilaments and silence of the TUFT1 could decrease the percentage of cells with actin cores in A549 cells, which is similar to the effect of wiskostatin, a selective N-WASP inhibitor which can bind the GTPase binding domain stabilizing the autoinhibited conformation (Fig. 6c, d). Over-expressed TUFT1 led to nucleus-translocation of p^Y256^N-WASP in MRC-5 cells, whereas the Wiskostatin could reverse this phenomenon completely (Additional file 1: Fig. S5). Meanwhile, TUFT1 enhanced the contractility of MRC-5 cells, whereas Wiskostatin inhibited the enhanced contractility induced by TUFT1 (Fig. 6e, f). Interestingly in MRC-5 cells, over-expressed TUFT1 induced a high level of p^Y256^N-WASP as well as FN, collagen I and α-SMA, while wiskostatin inhibited TUFT1 induced FN, collagen I, and α-SMA (Fig. 6g–k). Taken together, the above data illustrated that TUFT1 facilitates profibrotic responses in a p^Y256^N-WASP-dependent manner.

## Discussion

Repetitive injury to alveolar epithelial cells and activation of pulmonary fibroblasts are critical manifestations in the pathogenesis of IPF [30], and a previous study suggested that TUFT1 was inferred as a novel candidate gene for IPF [10]. In both IPF patients and a murine model of bleomycin-induced lung fibrosis, we observed a significant upregulation of TUFT1. Our investigation delved into the role that TUFT1 plays in experimental lung fibrosis in mice. Genetic silencing of TUFT1 mitigated bleomycin-induced lung fibrosis and concurrently reduced the bleomycin-induced increase in F-actin in mice. We found a positive correlation between F-actin expression and the extent of lung fibrosis, with both showing consistent alterations with TUFT1. The benefits observed in reducing lung fibrosis in mice via TUFT1 interference are likely associated with its impact on F-actin expression, primarily involving the assembly process from G-actin to F-actin.

Bundles of F-actin and DNA are pivotal constituents in the sputum of cystic fibrosis patients, capable of altering the viscoelastic properties of sputum, inhibiting its clearance, and exacerbating the progression of cystic fibrosis [31]. Studies have indicated that in terms of treatment, F-actin not only fails to enhance the barrier function of cystic fibrosis sputum but also significantly reduces the diffusion capability of mucolytics, further worsening the condition of cystic fibrosis patients [32]. Likewise, in research on interstitial pulmonary fibrosis, epithelial cells treated with TGF-β1 exhibit a notable increase in F-actin which is a hallmark of mesenchymal cells [33]. Hence, it is apparent that F-actin may play a critical role in pulmonary fibrosis. In this study, TUFT1 significantly influenced F-actin formation in both epithelium and fibroblasts cells, demonstrating a consistent relationship with the phenotypic changes towards a profibrotic state. These evidences suggest that TUFT1 likely affects pulmonary fibrosis by influencing the assembly of F-actin.

N-WASP plays a critical role in the formation of smooth muscle actin filaments during myofibroblast differentiation [24]. N-WASP is a significant regulator of the cell cytoskeleton, primarily modulating the assembly of F-actin by stimulating Arp2/3-mediated actin nucleation [34]. Moreover, research suggests that N-WASP can promote lung edema in acute lung injury mediated by TGF-β1 by regulating the dynamics of the actin cytoskeleton [35]. These data suggest the regulatory role of N-WASP likely extends to the early stages of lung fibrosis. Additionally, studies have shown that the Fyn/FAK/N-WASP pathway plays a crucial role in liver fibrosis, and inhibiting this pathway can provide significant benefits to patients with liver fibrosis [36]. These pieces of evidence indicate that N-WASP can influence the progression of fibrotic diseases through the assembly of F-actin and may serve as an intervention point for the treatment of fibrotic diseases.

Research has demonstrated that TUFT1 can be induced to express as a direct target of TGF-β1, and TUFT1 can influence the formation of F-actin in lung adenocarcinoma cells [16]. Our study reveals that TUFT1 can interact with N-WASP to promote the phosphorylation level of N-WASP. Furthermore, TUFT1 affects the intracellular distribution of p^Y256^N-WASP. These findings provide insights into the mechanism by which TUFT1 affects the formation of F-actin and establish a theoretical basis for understanding the role of TUFT1 in the process of pulmonary fibrosis. They also highlight the potential for further exploration of anti-fibrotic therapeutic drugs targeting N-WASP.

It is essential to acknowledge that the in vivo research conducted in this study utilized the single-dose bleomycin mouse model, which is the most widely adopted model for investigating IPF [37]. This model closely recapitulates various pathological processes and characteristics observed in IPF. However, due to our limited understanding of the disease mechanism and inherent species differences between mice and humans, this model cannot fully replicate IPF. It's essential to acknowledge that many treatments tested in this model have faced challenges in progressing beyond pre-clinical or clinical development stages, mainly due to imperfections in the model. Moreover, the underlying mechanism of the effects of TUFT1 on the phosphorylation of N-WASP is not fully unveiled and deserves further investigation. In addition, in vitro study was conducted on MRC-5 cell lines. It is important to note that MRC-5 cells are derived from embryonic lung tissue, and their characteristics may not entirely mimic the behavior and responses of primary human lung fibroblasts and may not fully capture the diversity of IPF phenotypes.

**Fig. 7:**
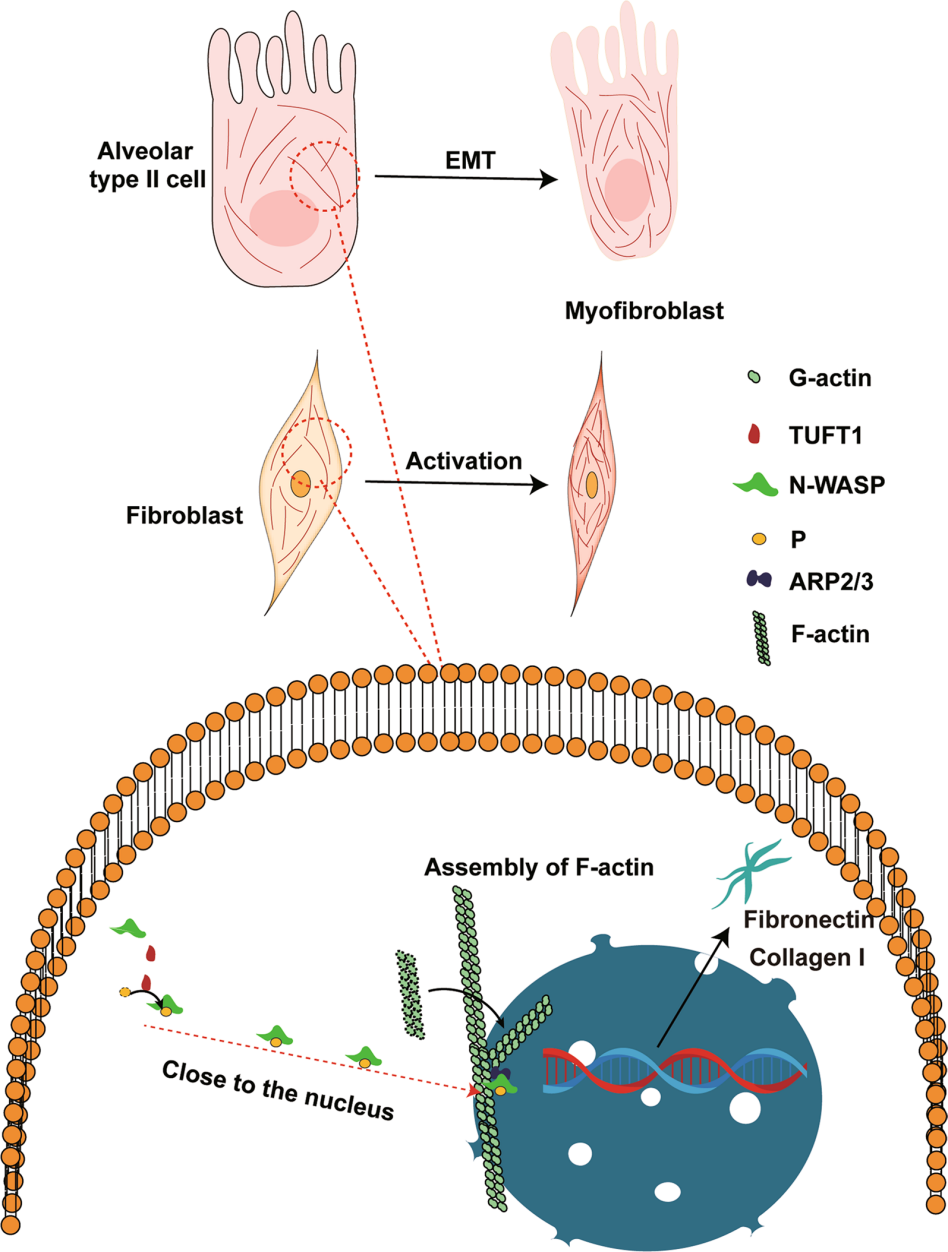
Schematic diagram shows TUFT1 Involvement in the Pathophysiological Processes of Pulmonary Fibrosis. Our study has unveiled a schematic model elucidating the role of TUFT1 in the pathophysiological processes underlying pulmonary fibrosis. Specifically, our data demonstrate that TUFT1 orchestrates the translocation of p^Y256^N-WASP into the cell nucleus, which facilitates the assembly of actin filaments, culminating in the enhanced formation of stress fibers. This heightened TUFT1-induced stress fiber formation appears to drive the cellular phenotypic transition toward a pro-fibrotic state

Our study has elucidated that TUFT1 exerts its pro-fibrotic effects by influencing the assembly of F-actin through N-WASP. This research marks a preliminary foray into understanding the role of TUFT1 in fibrotic diseases, introduces a novel pathogenic mechanism in the realm of IPF research, and evaluates the potential of N-WASP-F-actin as a therapeutic target for combating pulmonary fibrosis. Subsequent investigations should focus on delineating the specific mechanisms by which TUFT1 modulates p^Y256^N-WASP and validating the anti-fibrotic efficacy of Wiskostatin in vivo. Furthermore, exploring therapeutic strategies related to the N-WASP-F-actin pathway for anti-fibrotic interventions holds promise (Fig. 7).

## Conclusions

In conclusion, our data suggested that TUFT1 exerts pro-fibrotic effects by influencing stress fiber formation via promoting the phosphorylation of N-WASP in both epithelial and fibroblast cells. Given these findings, TUFT1 represents a promising therapeutic target for the attenuation of pulmonary fibrosis. Future research aimed at developing interventions targeting TUFT1 may hold the key to more effective treatments for this devastating lung condition.

### Supplementary Information

**Additional file 1: Figure S1.** Hologram of the Masson’s trichrome staining. The whole slides scan of four groups detected by H&E staining, scale bars: 4 mm. **Figure S2.** F-actin staining in bleomycin-challenge lung tissues. Phalloidin staining showed F-actin was abundant in fibrotic tissues of bleomycin-induced lung fibrosis, whereas Tuft1/shRNA decreased F-actin formation in bleomycin challenged lung tissues, scale bars: 200 µm. **Figure S3.** TUFT1 interacted with N-WASP and affected the expression of p^Y256^N-WASP. (A) N-WASP and TUFT1 were co-localized in A549 cells detected by immunofluorescent staining, scale bars: 10 µm. (B) TUFT1 can induce a high expression of p^Y256^N-WASP in A549 cells. (C) Silencing the TUFT1 could make the p^Y256^N-WASP keep away from the nucleus compared to the control detected by immunofluorescent staining, scale bars: 20 µm. (D) The distance of p^Y256^N-WASP to the core of the nucleus was quantified. **Figure S4.** Silencing the Tuft1 could disperse the p^Y256^N-Wasp in the fibrotic areas. The expression of p^Y256^N-Wasp in mice model was detected by immunohistochemistry staining, scale bars: 200 µm. **Figure S5.** The localization of p^Y256^N-WASP in MRC5 cells. Over-expressed TUFT1 changed p^Y256^N-WASP distribution close to the nucleus in MRC5 cells, whereas the Wiskostatin could reverse this phenomenon completely, scale bars: 100 µm.

## Data Availability

The cells used during the current study are available from the corresponding author on reasonable request.

## Abbreviations

IPF: Idiopathic pulmonary fibrosis
ILD: Interstitial lung disease
BLM: Bleomycin
TUFT1: Tuftelin1
N-WASP: Neuronal Wiskott-Aldrich syndrome protein
TGF-β1: Transforming growth factor-β1
p^Y256^N-WASP: The phosphorylation of tyrosine residue 256 (Y256) of the N-WASP
Wis: Wiskostatin
SLB: Surgical lung biopsy
SOV: Sodium orthovanadate
α-SMA: Alpha-smooth muscle actin
