# Supplementary material for: Tuftelin1 drives experimental pulmonary fibrosis progression by facilitating stress fiber assembly
Source: Respir Res. 2023 Dec 17;24:318. doi: 10.1186/s12931-023-02633-w (PMC10726504; doi:10.1186/s12931-023-02633-w)
Supplement: Supplementary file 1 — Additional file 1: Figure S1. Hologram of the Masson’s trichrome staining. The whole slides scan of four groups detected by H&E staining, scale bars: 4 mm. Figure S2. F-actin staining in bleomycin-challenge lung tissues. Phalloidin staining showed F-actin was abundant in fibrotic tissues of bleomycin-induced lung fibrosis, whereas Tuft1/shRNA decreased F-actin formation in bleomycin challenged lung tissues, scale bars: 200 µm. Figure S3. TUFT1 interacted with N-WASP and affected the expression of pY256N-WASP. (A) N-WASP and TUFT1 were co-localized in A549 cells detected by immunofluorescent staining, scale bars: 10 µm. (B) TUFT1 can induce a high expression of pY256N-WASP in A549 cells. (C) Silencing the TUFT1 could make the pY256N-WASP keep away from the nucleus compared to the control detected by immunofluorescent staining, scale bars: 20 µm. (D) The distance of pY256N-WASP to the core of the nucleus was quantified. Figure S4. Silencing the Tuft1 could disperse the pY256N-Wasp in the fibrotic areas. The expression of pY256N-Wasp in mice model was detected by immunohistochemistry staining, scale bars: 200 µm. Figure S5. The localization of pY256N-WASP in MRC5 cells. Over-expressed TUFT1 changed pY256N-WASP distribution close to the nucleus in MRC5 cells, whereas the Wiskostatin could reverse this phenomenon completely, scale bars: 100 µm. [file 12931_2023_2633_MOESM1_ESM.docx]

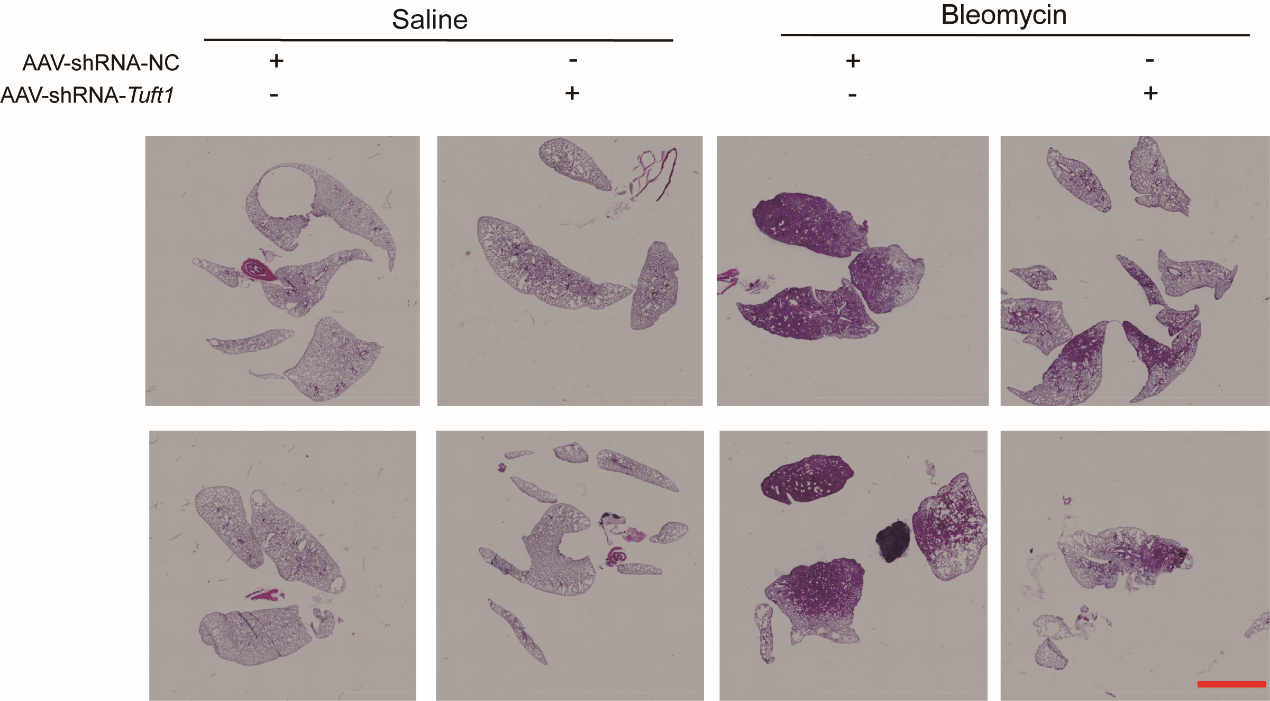


**Figure S1 Hologram of the Masson’s trichrome staining.** The whole slides scan of four groups detected by H&E staining, scale bars: 4mm.


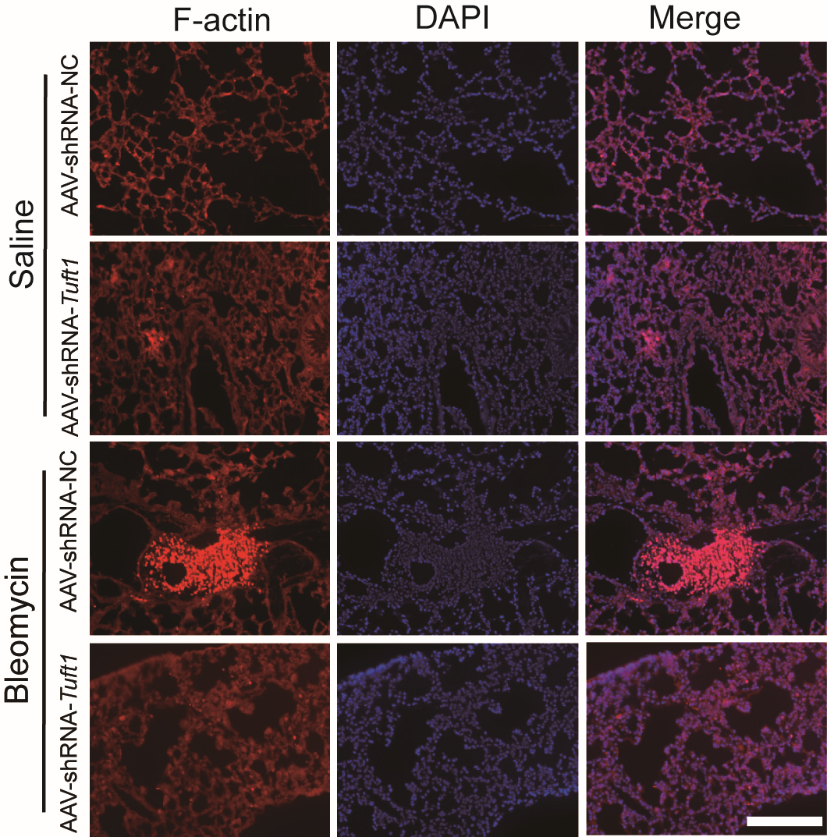


**Figure S2 F-actin staining in bleomycin-challenge lung tissues.** Phalloidin staining showed F-actin was abundant in fibrotic tissues of bleomycin-induced lung fibrosis, whereas Tuft1/shRNA decreased F-actin formation in bleomycin challenged lung tissues, scale bars: 200µm.


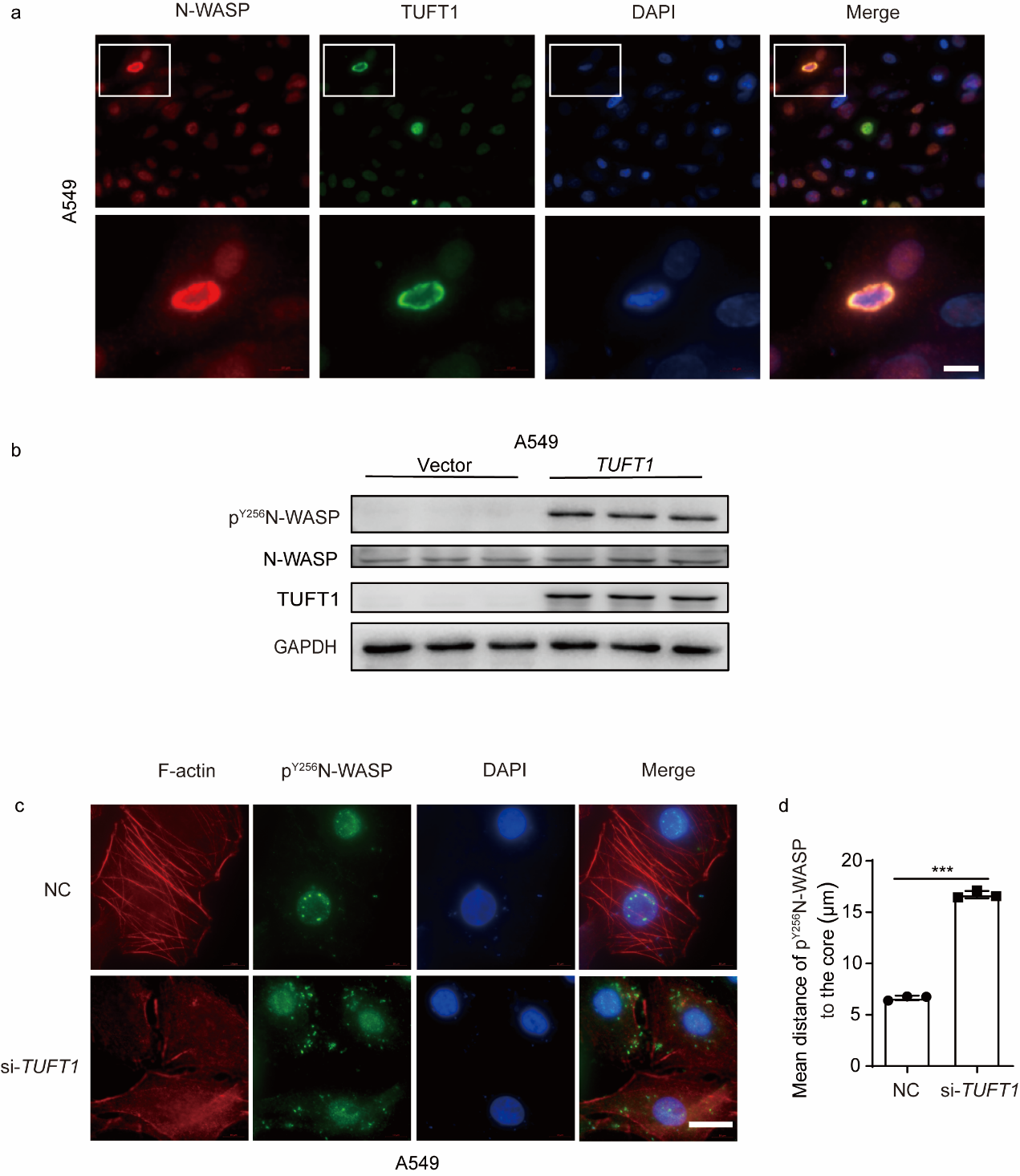


**Figure S3 TUFT1 interacted with N-WASP and affected the expression of p^Y256^N-WASP.** (a) N-WASP and TUFT1 were co-localized in A549 cells detected by immunofluorescent staining, scale bars: 10 µm. (b) TUFT1 can induce a high expression of p^Y256^N-WASP in A549 cells. (c) Silencing the TUFT1 could make the p^Y256^N-WASP keep away from the nucleus compared to the control detected by immunofluorescent staining, scale bars: 20 µm. (d) The distance of p^Y256^N-WASP to the core of the nucleus was quantified.


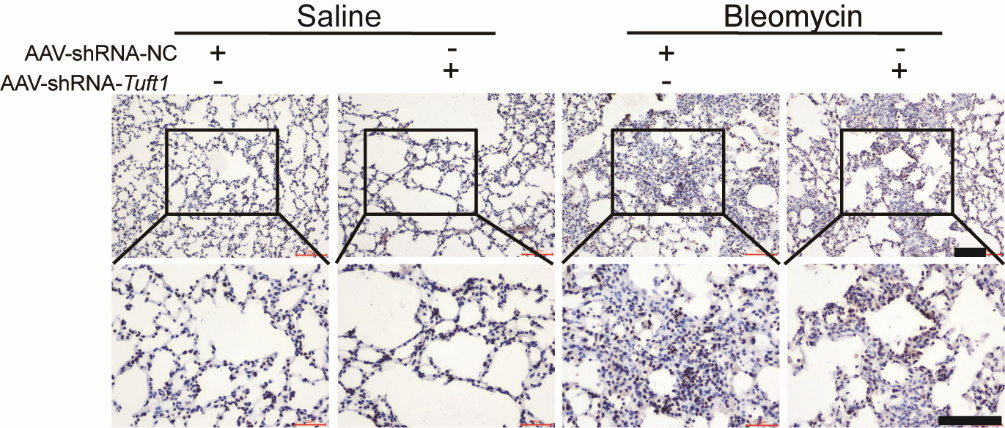


**Figure S4 Silencing the Tuft1 could disperse the p^Y256^N-Wasp in the fibrotic areas.** The expression of p^Y256^N-Wasp in mice model was detected by immunohistochemistry staining, scale bars: 200 µm.


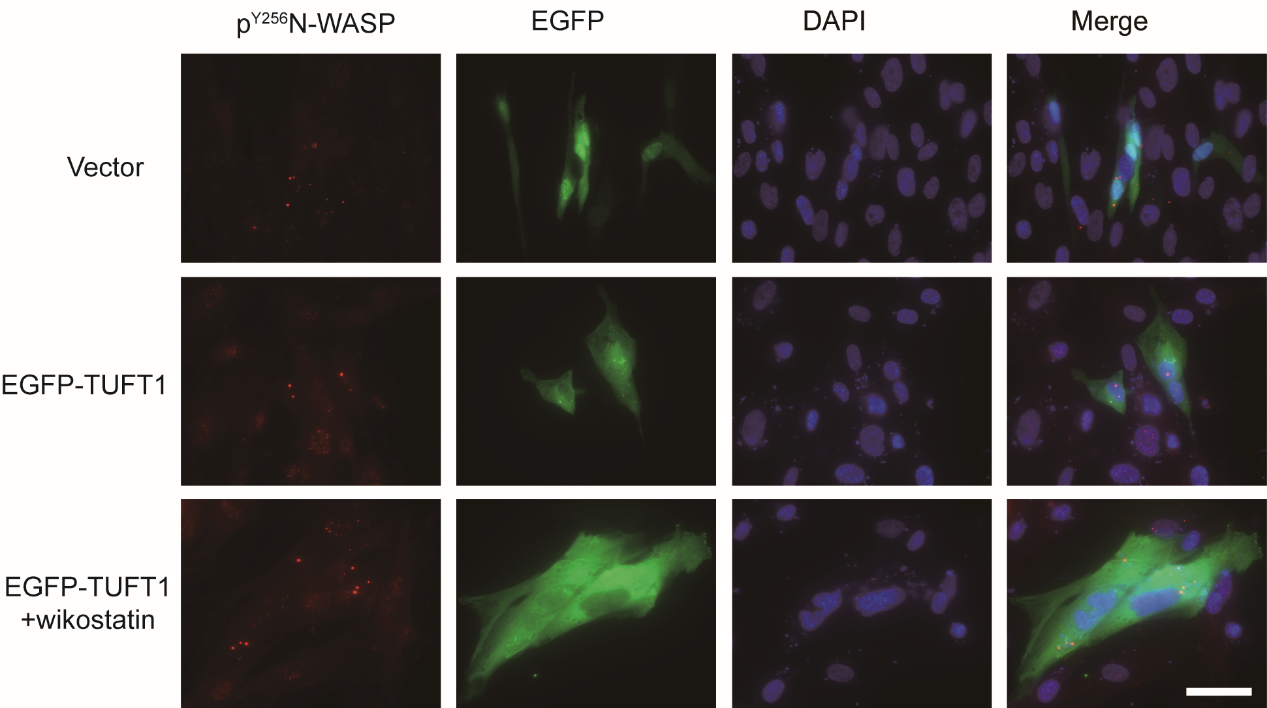


**Figure S5 The localization of p^Y256^N-WASP in MRC5 cells.** Over-expressed TUFT1 changed p^Y256^N-WASP distribution close to the nucleus in MRC5 cells, whereas the Wiskostatin could reverse this phenomenon completely, scale bars: 100 µm.
